# Systematic Review of Resection Rates and Clinical Outcomes After FOLFIRINOX-Based Treatment in Patients with Locally Advanced Pancreatic Cancer

**DOI:** 10.1245/s10434-016-5373-2

**Published:** 2016-07-01

**Authors:** Steffi J. Rombouts, Marieke S. Walma, Jantien A. Vogel, Lennart B. van Rijssen, Johanna W. Wilmink, Nadia Haj Mohammad, Hjalmar C. van Santvoort, I. Quintus Molenaar, Marc G. Besselink

**Affiliations:** 1 Department of Surgery, University Medical Centre Utrecht Cancer Center, Utrecht, The Netherlands; 2 Department of Surgery, G4-196, Academic Medical Centre, Amsterdam, The Netherlands; 3 Department of Medical Oncology, Academic Medical Centre, Amsterdam, The Netherlands; 4 Department of Medical Oncology, University Medical Centre Utrecht Cancer Center, Utrecht, The Netherlands; 5 Department of Surgery, St. Antonius Hospital, Nieuwegein, The Netherlands

## Abstract

**Background:**

FOLFIRINOX prolongs survival in patients with metastatic pancreatic cancer and may also benefit patients with locally advanced pancreatic cancer (LAPC). Furthermore, it may downstage a proportion of LAPC into (borderline) resectable disease, however data are lacking. This review assessed outcomes after FOLFIRINOX-based therapy in LAPC.

**Methods:**

The PubMed, EMBASE and Cochrane library databases were systematically searched for studies published to 31 August 2015. Primary outcome was the (R0) resection rate.

**Results:**

Fourteen studies involving 365 patients with LAPC were included; three studies administered a modified FOLFIRINOX regimen. Of all patients, 57 % (*n* = 208) received radiotherapy. The pooled resection rate was 28 % (*n* = 103, 77 % R0), with a perioperative mortality of 3 % (*n* = 2), and median overall survival ranged from 8.9 to 25.0 months. Survival data after resection were scarce, with only one study reporting a median overall survival of 24.9 months in 28 patients. A complete pathologic response was found in 6 of 85 (7 %) resected specimens. Dose reductions were described in up to 65 % of patients, grade 3–4 toxicity occurred in 23 % (*n* = 51) of patients, and 2 % (*n* = 5) had to discontinue treatment. Data of patients treated solely with FOLFIRINOX, without additional radiotherapy, were available from 292 patients: resection rate was 12 % (*n* = 29, 70 % R0), with 15.7 months median overall survival and 19 % (*n* = 34) grade 3–4 toxicity.

**Conclusions:**

Outcomes after FOLFIRINOX-based therapy in patients with LAPC seem very promising but further prospective studies are needed, especially with regard to survival after resection.

Pancreatic ductal adenocarcinoma has very poor survival rates. Surgical resection with adjuvant chemotherapy offers the best survival but is only feasible in approximately 20 % of patients.^1^ Forty percent of patients present without distant metastases but with extensive vascular involvement prohibiting upfront resection, known as locally advanced pancreatic cancer (LAPC).^1^ In these patients, gemcitabine monotherapy (sometimes combined with radiotherapy) has been the standard palliative treatment for decades. Unfortunately, response rates are low without clear improvement in survival.^2^

Recently, the superiority of FOLFIRINOX, a combination of 5-fluorouracil, oxaliplatin, irinotecan and leucovorin, over gemcitabine monotherapy in patients with metastatic pancreatic cancer was demonstrated: a response rate of 31.6 versus 9.4 % and a median overall survival of 11.1 months versus 6.8 months (*p* < 0.001) has been observed.^3^ The comparable poor prognosis of LAPC and the lack of beneficial therapies have also led to the administration of FOLFIRINOX, sometimes combined with radiotherapy, in patients with LAPC; however, no randomized trials have been conducted on this topic.

Several observational studies on FOLFIRINOX-based treatment included both patients with LAPC and borderline resectable pancreatic cancer. Borderline resectable disease is defined by the National Comprehensive Cancer Network (NCCN) as an arterial involvement of less than 180 degrees or a venous involvement with options for reconstruction.^4^ The inclusion of patients with borderline resectable pancreatic cancer may positively influence outcomes as these patients have a higher chance of resection in advance. Therefore, the aim of this study was to evaluate the results of FOLFIRINOX-based treatment only in patients with LAPC, considering (R0) resection rate as the primary outcome.

## Methods

This systematic review was performed according to the Preferred Reporting Items for Systematic Reviews and Meta-Analyses (PRISMA) guidelines.^5^

### Search and Selection

The PubMed, EMBASE, and Cochrane Library databases were systematically searched for studies published from 2005 to 31 August 2015. Duplicates were removed and studies published in languages other than English were excluded. Three authors (MW, SR, JV) independently screened articles by title and abstract and, if applicable, the full articles for eligibility based on predefined inclusion and exclusion criteria. Discordant judgments were addressed by consulting a fourth author (LR). The reference lists of all included papers were searched manually to identify missed, but potentially relevant, studies.

### Eligibility Criteria

Retrospective and prospective studies on FOLFIRINOX in patients with LAPC, reporting (R0) resection rate, survival, response rate or toxicity, were eligible for inclusion in our study. Conference abstracts or case reports (i.e. sample size of fewer than five patients) were excluded.

### Assessment of Methodological Quality

The level of evidence was classified and a classical risk of bias assessment was applied for all included studies according to the Oxford Centre for Evidence-Based Medicine (CEBM) Critical Appraisal Skills Programme (CASP) 2004.^6^,^7^

### Data Collection

Study design, study population, sample size, resectability criteria and treatment regimen were extracted from the included studies. Primary outcome was the (R0) resection rate. Secondary outcomes were postoperative complications, pathological response, overall survival, response rate, CA19-9 response, and toxicity. In addition, if FOLFIRINOX treatment was followed by radiotherapy, outcomes during FOLFIRINOX administration before the start of radiotherapy were additionally extracted to get more insight into the outcome for solely FOLFIRINOX treatment. Corresponding authors were approached when data were missing or could not be extracted from the article, or if no data were presented for the LAPC population separately.

### Statistical Analysis

Overall (R0) resection rate, postoperative complications, complete pathologic response, response rate, CA19-9 response, and toxicity were calculated. A meta-analysis of overall survival was not performed because of substantial heterogeneity between studies and lack of individual patient data.

## Results

**Fig. 1:**
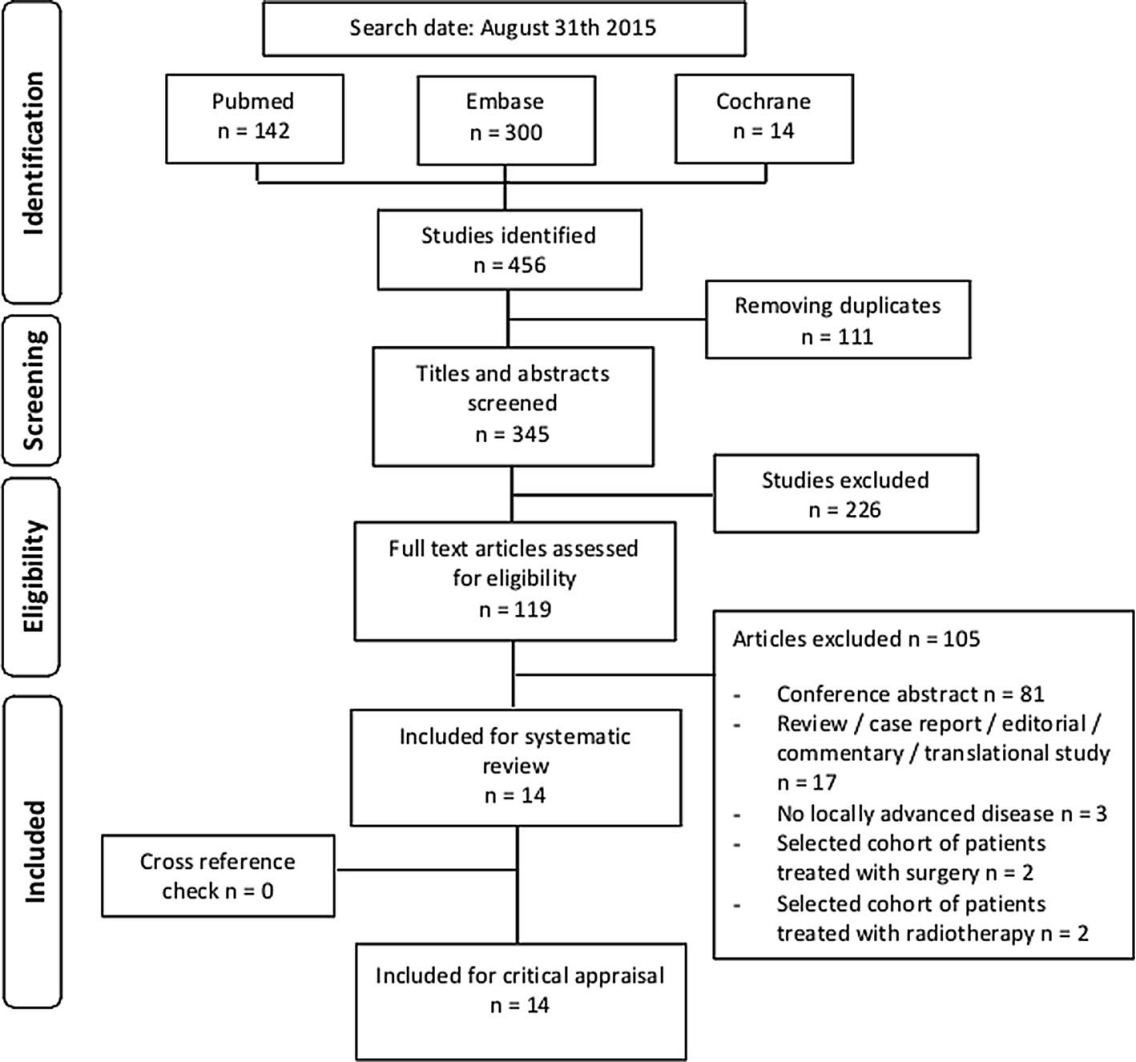
Study selection process

Fourteen studies involving 365 patients (one prospective observational study 10 and 13 retrospective studies 8,9,11–21) were included (Fig. 1). No randomized trials were available. LAPC was defined according to the National Comprehensive Cancer Network (NCCN) (*n* = 4),^4^,^11^,^12^,^18^,^21^ the consensus statement of the American Hepato-Pancreato-Biliary Association [AHPBA/SSAT/SSO] (*n* = 3),^8^,^9^,^14^,^22^ or based on consensus within the multidisciplinary team (*n* = 2).^10^,^17^ Five articles did not define LAPC,^13^,^15^,^16^,^19^,^20^ and all studies had a substantial risk of bias (Table 1).

**Table 1:** Risk of bias assessment

Author	Level of evidence^a^	Criteria for LAPC	Clear inclusion and exclusion criteria	Risk of selection bias	Standardized intervention	Standardized treatment^b^	Standardized outcome	Confounding factors	Missing data verified	Duration of follow-up >12 months	Lost to follow-up
Blazer et al.^8^	2b	AHPBA/SSO/SSAT	+	+	+	+	±	+	+	+	+
Boone et al.^9^	2b	AHPBA/SSO/SSAT	+	+	NR	±	±	+	NR	−	+
Conroy et al.^10^	2b	MDT	±	±	+	±	+	+	+	+	NR
Faris et al.^11^	2b	NCCN	+	+	+	±	±	+	+	+	NR
Gunturu et al.^12^	2b	NCCN	+	+	+	±	±	+	NR	+	NR
Hohla et al.^13^	2b	NR	±	+	+	±	±	+	NR	−	NR
Hosein et al.^14^	2b	AHPBA/SSO/SSAT	+	+	+	+	±	+	NR	+	NR
Kraemer et al.^15^	2b	NR	±	+	+	−	−	+	NR	−	NR
Mahaseth et al.^16^	2b	NR	±	+	+	−	+	±	NR	+	+
Marthey et al.^17^	2b	MDT	±	+	+	±	+	±	NR	+	NR
Mellon et al.^18^	2b	NCCN	+	+	+	+	+	NR	+	−	NR
Moorcraft et al.^19^	2b	NR	±	+	+	−	+	±	NR	+	NR
Peddi et al.^20^	2b	NR	±	+	+	−	+	±	+	+	NR
Sadot et al.^21^	2b	NCCN	+	+	+	+	+	±	+	+	NR

*LAPC* locally advanced pancreatic cancer, *NR* not reported, *AHPBA/SSO/SSAT* American Hepato-Pancreato-Biliary Association/Society of Surgical Oncology/Society for Surgery of the Alimentary Tract^22^, *CEBM* Centre for Evidence-Based Medicine, *MDT* multidisciplinary team, *NCCN* National Comprehensive Cancer Network^4^, + indicates yes, − indicates no, ± indicates partially

^a^According to the Oxford CEBM levels of evidence^6^

^b^With regard to pre- and post-interventional treatments

### Treatment Regimen

FOLFIRINOX was administered as single treatment in four studies 10,12,13,15 and combined with radiotherapy in 10 studies.^8^,^9^,^11^,^14^,^16^–^21^ Three of the 14 studies administered a modified FOLFIRINOX regimen from the beginning of therapy by eliminating the bolus of fluorouracil,^16^ in addition to lowering the dose of irinotecan,^8^ or by a starting dose of 80 % of the intensity of the FOLFIRINOX regimen.^21^ One study administered a modified regimen in 68 % of all first cycles.^20^ In the remaining 10 studies, FOLFIRINOX was administered as per the PRODIGE 4/ACCORD 11 trial protocol at the start, but also reported on a dose-reduction during the course of treatment for 63 % of total cycles and in up to 65 % of patients.^3^,^9^–^15^,^17^–^19^ The median number of cycles was reported in five studies and ranged from four to eight.^8^,^11^,^17^,^20^,^21^ Five studies reported FOLFIRINOX to be first-line treatment.^12^,^14^,^15^,^17^,^21^ Patients who had progression under FOLFIRINOX treatment and subsequent radiotherapy were treated with second-line chemotherapy in two studies.^17^,^21^ After resection, adjuvant gemcitabine-based chemotherapy was reported by two studies, as well as additional combined chemoradiotherapy by one study.^8^,^14^,^15^ The remaining seven studies did not reported on prior, second-line, or adjuvant therapy.^9^,^10^,^13^,^16^,^18^–^20^

Overall, 208 of 362 patients (57 %) were treated with additional radiotherapy after FOLFIRINOX treatment (Table 2). Radiotherapy was delivered through conventional treatment,^8^,^11^,^14^ intensity-modulated radiation therapy (IMRT),^16^,^17^,^19^,^21^ or as stereotactic body radiation therapy (SBRT).^9^,^18^ One study did not report on the details of radiotherapy.^20^ Radiation was combined with chemotherapy in six studies.^8^,^11^,^14^,^16^,^19^,^21^ The chemosensitizer, as part of the chemoradiation, differed between gemcitabine, capecitabine, 5-fluorouracil, or a combination. The total administered dose of radiotherapy ranged from 36 to 54 Gy, given in fractions ranging from 3 to 30. Three studies did not report on the dosage of radiotherapy.^16^,^20^,^21^

**Table 2:** Outcomes after FOLFIRINOX-based treatment in patients with LAPC

Author	No. of patients	Treated with radiotherapy	Resection rate	R0 resection rate	Complete pathologic response	Response rate	Median OS (months)	Grade 3–4 toxicity
Blazer et al.^8^	25	15/25 (60)	11/25 (44)	10/11 (91)	0/11 (0)	2/23 (9)^a^	NR	NR
Boone et al.^9^	13^b^	5/10 (50)	2/10 (20)	1/2 (50)	NR	NR	8.9	5/10 (50)
Conroy et al.^10^	11^c^	0 (0)	0/11 (0)	NA	NA	3/11 (27)	15.7	NR
Faris et al.^11^	22	20/22 (91)	5/22 (23)	5/5 (100)	1/5 (20)	8/22 (36)	NRE, 3-year 7 %	NR
Gunturu et al.^12^	16	0 (0)	2/16 (13)	NR	0/2 (0)	8/16 (50)	NRE, 6-month 94 %; 12-month 83 %	NR
Hohla et al.^13^	6	0 (0)	2/6 (33)	NR	NR	NR	NR	NR
Hosein et al.^14^	14	9/14 (64)	6/14 (43)	5/6 (83)	NR	NR	NR	NR
Kraemer et al.^15^	7	0 (0)	1/7 (14)	0/1 (0)	0/1 (0)	NR	NR	NR
Mahaseth et al.^16^	20	10/20 (50)	4/20 (20)	3/4 (75)	NR	NR	NR	NR
Marthey et al.^17^	77	54/77 (70)	28/77 (36)	25/28 (89)	4/28 (14)	22/77 (28)	21.6	20/77 (26)
Mellon et al.^18^	21	21/21 (100)	5/21 (24)	5/5 (100)	0/5 (0)	NR	NR	NR
Moorcraft et al.^19^	13	7/13 (54)	2/13 (15)	2/2 (100)	1/2 (50)	4/13 (31)	18.4	7/13 (54)
Peddi et al.^20^	19	4/19 (21)	4/19 (21)	NR	NR	NR	NRE	5/19 (26)
Sadot et al.^21^	101	63/101 (62)	31/101 (31)	16/29 (55)^d^	0/31 (0)	29/101 (29)	25	14/101 (14)
Overall	365	208/362 (57)	103/362 (28)	72/93 (77)	6/85 (7)	76/263 (29)		51/220 (23)

Data are expressed as *n* (%) unless otherwise specified

*OS* overall survival, *NA* not applicable, *NR* not reported, *NRE* not reached, *LAPC* locally advanced pancreatic cancer

^a^Two patients died before the restaging scan

^b^Three patients refused treatment or were lost to follow-up

^c^One patient had a local recurrence

^d^Two pathology reports were pending

### Resection Rate and Postoperative Outcomes

Each of the 14 studies reported on resection rates, with a total of 28 % (*n* = 103) after a median of five to eight cycles of FOLFIRINOX and additional radiotherapy in 66 % of patients (56 of 85 patients with available data) (Table 2).^9^,^10^,^12^–^18^,^20^,^21^ Of these, 10 studies reported a total R0 resection rate of 77 % (*n* = 72).^8^,^9^,^11^,^14^–^19^,^21^ Morbidity after resection was reported in three studies including 64 patients, and ranged from 20 % grade 3–4 to 60 % overall complications.^11^,^17^,^21^ Morbidity was specified for 33 patients, with postoperative infection (*n* = 5) and bleeding (*n* = 3) as the most common cause. Pancreatic fistula was reported in one patient, and median hospital stay ranged from 6 to 7 days.^11^,^21^ Perioperative mortality, reported by five studies, was 3 % (*n* = 2).^8^,^9^,^17^,^18^,^21^ In total, 6 of 85 (7 %) resection specimens showed a complete pathologic response (Table 2).^8^,^11^,^12^,^15^,^17^–^19^,^21^

One study compared patients who proceeded to surgery with those who did not (*n* = 31 and *n* = 70, respectively). Hepatic artery and unreconstructable venous involvement were more common in the group that proceeded to resection compared with celiac trunk, superior mesenteric artery, or multiple vessel involvement (*p* = 0.001).^21^ Another study did not reach significance when comparing arterial involvement with venous involvement in resected patients.^17^ No studies specified vascular involvement in degrees.

Eight studies reported the resection rate for solely FOLFIRINOX treatment, without additional radiotherapy, with a pooled resection rate of 12 % (*n* = 29).^9^,^10^,^12^–^15^,^17^,^21^ In addition, four of these studies reported 14 R0 resections (70 %) from a total of 20 resections 9,14,15,21 without any complete pathologic response (Table 3).

**Table 3:** Outcomes of solely FOLFIRINOX treatment, including studies reporting data after FOLFIRINOX treatment prior to the start of additional radiotherapy

Author	No. of patients	Resection rate	R0 resection rate	Complete pathologic response	Response rate	Median OS (months)	Grade 3–4 toxicity
Blazer et al.^8^	25	–	–	–	2/23 (9)^a^	NR	NR
Boone et al.^9^	13^b^	2/10 (20)	1/2 (50)	NR	NR	NR	–
Conroy et al.^10^	11^c^	0/11 (0)	NA	NA	3/11 (27)	15.7	NR
Faris et al.^11^	22	–	–	–	6/22 (27)	–	NR
Gunturu et al.^12^	16	2/16 (13)	NR	0/2 (0)	8/16 (50)	NRE, 6-month 94 %; 12-month 83 %	NR
Hohla et al.^13^	6	2/6 (33)	NR	NR	NR	NR	NR
Hosein et al.^14^	14	3/14 (21)	2/3 (67)	NR	NR	NR	–
Kraemer et al.^15^	7	1/7 (14)	0/1 (0)	0/1 (0)	NR	NR	NR
Marthey et al.^17^	77	4/77 (5)	–	–	–	–	20/77 (26)
Sadot et al.^21^	101	15/101 (15)	11/14 (79)^d^	0/15 (0)	20/101 (20)	–	14/101 (14)
Overall	292	29/242 (12)	14/20 (70)	0/18 (0)	39/173 (23)		34/178 (19)

Data are expressed as *n* (%) unless otherwise specified

*OS* overall survival, *NR* not reported, *NA* not applicable, *NRE* not reached, – indicates not reported separately for FOLFIRINOX, only combined with radiotherapy (Table 2)

^a^Two patients died before the restaging scan

^b^Three patients refused treatment or were lost to follow-up

^c^One patient had a local recurrence

^d^One pathology report was pending

### Median Overall Survival

The median overall survival was reported in five studies and ranged from 8.9 to 25 months; of these patients, 64 % were treated with radiotherapy.^9^,^10^,^17^,^19^,^21^ In three studies, median survival was not reached.^11^,^12^,^20^ One study showed a 1-year survival of 83 %, in which the majority of patients (91 %) were treated with radiotherapy.^12^ A second study showed a 3-year survival of 7 %; none of the patients received radiotherapy (Table 2).^11^

In addition, one study reported a median overall survival of 24.9 months in 28 patients who underwent pancreatic resection.^17^ Resection was preceded by radiotherapy in 24 patients. In two other studies, survival data after resection were available from only two patients.^9^,^19^ Only one study treated LAPC patients with solely FOLFIRINOX, without additional radiotherapy or resection, and reported a median overall survival of 15.7 months (Table 3).^10^

### Response Rate and CA-19.9 Response

Seven studies reported on response rates.^8^,^10^–^12^,^17^,^19^,^21^ Almost all defined response rate as complete or partial response according to Response Evaluation Criteria In Solid Tumors (RECIST) criteria,^11^–^13^,^16^,^17^,^19^–^21^ and one according to the World Health Organization (WHO) criteria.^10^ Of the 238 patients who were treated with FOLFIRINOX, 67 % of patients received additional radiotherapy, which led to response rates ranging from 9 % (*n* = 2) to 50 % (*n* = 8), with a total response rate of 29 % (*n* = 76) (Table 2). CA-19.9 reduction was reported in three studies: an overall >30 % reduction in 70 % of patients, an overall >50 % reduction in 54 % of patients, and a normalization of the concentration in 35 % of all patients.^8^,^11^,^17^

In case of solely FOLFIRINOX treatment, response rates ranged from 9 % (*n* = 2) to 50 % (*n* = 8), with a total of 23 % (*n* = 39) (Table 3). Three studies that administered subsequent radiotherapy in selected patients reported response rates before and after radiotherapy, and showed an additional response ranging from 0 % (*n* = 0) to 9 % (*n* = 9) due to radiotherapy treatment.^8^,^11^,^21^

### Toxicity

Five studies reported a 23 % (*n* = 51) grade 3–4 toxicity, without grade 5 toxicity (Table 2).^9^,^17^,^19^–^21^ None of the studies reported specifically on the toxicity caused by radiation. When considering toxicity for FOLFIRINOX alone, two studies reported a total grade 3–4 toxicity rate of 19 % (*n* = 34) and no grade 5 toxicity (death) (Table 3).^17^,^21^ The most common grade 3 and 4 complications were neutropenia (10 %) and nausea or vomiting (9 %).^9^,^17^,^19^,^20^ Eight studies reported on discontinuation of treatment due to unacceptable toxicity, with a pooled discontinuation rate of 2 % (*n* = 5).^10^,^11^,^14^,^16^–^18^,^21^

## Discussion

This systematic review on clinical outcomes after FOLFIRINOX-based treatment for LAPC demonstrated a 28 % resection rate, of which 77 % were R0, and a median overall survival ranging between 8.9 and 25.0 months. Fifty-seven percent of these patients were treated with additional radiotherapy. These data suggest that FOLFIRINOX-based treatment is indeed a promising option for patients with LAPC, with acceptable toxicity (23 % grade 3–4 complications). After surgical resection, survival data were lacking as only one study reported a median overall survival of 24.9 months.^17^

One previous review included studies published up to March 2014 and reported resection rates from six studies.^23^ The current review, including 14 studies, gives an updated overview and shows other clinical outcomes after FOLFIRINOX treatment specifically in patients with LAPC. As expected, the overall R0 resection rates reported in our review (70–77 %) are slightly lower, as reported by two recent studies (84–92 %) on borderline resectable disease.^24^,^25^ Surgical outcomes post-resection seem comparable with outcomes in upfront resectable patients, although still based on immature data.^26^–^28^

Although no study directly compared outcomes after FOLFIRINOX versus gemcitabine monotherapy in LAPC, the results of FOLFIRINOX seem clearly superior to gemcitabine, with reported response rates of 4.2–14.9 % and a resection rate of only 7 %.^29^,^30^ Moreover, none of the established therapies for LAPC have reported resection rates similar to those of FOLFIRINOX reported in our review.^31^

When addressing toxicity, our review shows remarkable lower toxicity rates compared with the PRODIGE 4/ACCORD 11 trial,^3^ which reported 46 % grade 3–4 neutropenia compared with 19 % after FOLFIRINOX alone in our review. In the PRODIGE/ACCORD trial, the median number of treatment cycles administered was 10 and the median relative dose intensities of fluorouracil, irinotecan, and oxaliplatin were 82, 81 and 78 %, respectively. This suggests that the reduced toxicity rate in our review is probably explained by the administered modified regimens by start and/or dose reductions during treatment, as described in all included studies.

Our study has some limitations. First, the allocation of FOLFIRINOX was often not based on predefined criteria but at the discretion of the treating team. Therefore it is inevitable that selection bias has occurred. No randomized trials are performed and all studies reported only on patients who actually received (or even completed) FOLFIRINOX treatment. In other words, the percentage of patients with LAPC not receiving FOLFIRINOX treatment and the survival in the entire cohort of LAPC were not reported. Furthermore, only half of the studies reported the guidelines used to establish resectability (Table 1). These guidelines use various definitions.^4^,^22^ Moreover, studies reporting on survival after resection with FOLFIRINOX in LAPC are scarce and immature. Finally, the interventional treatment was not standardized. Different dose reductions and modification schemes were applied and were not performed according to a protocolled reduction schedule, but based on the preference of the treating physician. In addition, the radiotherapy regimens varied between the studies.

An important clinical question is how to decide which patient may benefit from surgical exploration after FOLFIRINOX treatment. A recent study clearly demonstrated that post-FOLFIRINOX CT-based treatment decision making in pancreatic cancer is highly unreliable.^24^ In that study, a senior pancreatic surgeon, blinded to FOLFIRINOX treatment, judged 19 of the 40 resected patients as non-resectable based on post-FOLFIRINOX imaging; however, all 40 patients underwent a resection, with a remarkable 92 % R0 resection rate and a median overall survival of 35 months for the entire group (19 LAPC and 9 borderline). Several other studies have also recommended an exploratory laparotomy after induction therapy in the absence of disease progression on subsequent imaging.^32^,^33^ These new insights on the low accuracy of CT imaging in the assessment of resectability, and thus the recommendation for surgical exploration after induction, suggest that the resection rates demonstrated in these previously published studies might currently be even higher in expert centers. It is currently unclear whether a different approach should be taken in patients with LAPC compared with these series, which also included patients with borderline resectable disease. Future studies should validate selection criteria for surgical exploration. Improved imaging modalities are urgently needed to improve the post-FOLFIRINOX decision-making process.

This review demonstrates the need for prospective unselected studies with strict definitions, thus including patients not receiving FOLFIRINOX. Such studies should ideally report on consecutive patient (treatment) outcomes, including quality of life, and on the overall survival of all patients, especially those undergoing resection after FOLFIRINOX. Since a randomized controlled trial comparing FOLFIRINOX with gemcitabine for LAPC seems unethical, future prospective unselected cohort studies are recommended to investigate which patients might be eligible for, and could benefit from, FOLFIRINOX and/or multimodality treatments. Finally, studies should focus on optimizing selection criteria for surgical exploration after FOLFIRINOX in LAPC.^24^

## Conclusions

Outcomes after FOLFIRINOX treatment in patients with LAPC are promising, both for toxicity and (R0) resection rates. Future unselected prospective cohort studies are needed to determine the exact role for FOLFIRINOX in LAPC.
